# Misapprehensions *v*. Facts

**Published:** 1888-01-07

**Authors:** 


### MISAPPREHENSIONS v. FACTS.

Let us now take the errors set out above in the order in which they occur :—

1. *The Registration of Trained Nurses.*—The proposals to which the writer refers are those formulated by the Matrons' Sectional Committee of The Hospitals Association and its Council after meeting in conference some months ago. They have never been put in force, because it has been felt from the first that no attempt to establish a Register of Nurses could succeed unless it was based upon proposals agreed to by the representatives of the whole of the Nurse Training Schools. Those, however, who declare, if any such there be, that The Hospitals Association is desirous of establishing a Register of Nurses upon any such basis as our correspondent describes, state that which is not the fact. The Council of The Hospitals Association have decided to be no parties to any such proposals, nor will they support any scheme for the registration of nurses which has not first been agreed to by the representatives of the Nurse Training Schools. It is further to be noted that Mr. Burdett has proposed no scheme of any kind whatever for the registration of trained nurses, and has taken no part in the registration scheme, except so far as his being a member of the Council of the Hospitals Association makes him responsible for the action of that body, as explained already. "The point of issue" our correspondent alludes to can, under these circumstances, have therefore no existence in fact, and has never really existed, for the following further reasons.

2. *The National Pension Fund.*—This Fund has no connexion with the scheme for the registration of nurses, and never has had. All nurses who can prove themselves to be deservedly entitled to be so called will be eligible to join the Pension Fund. Married women, for reasons which the actuaries believe to be unanswerable, will probably not be eligible for the Sick Fund, but even they may become entitled to a pension subject to certain conditions to be set forth in the rules now in course of preparation. There is, therefore, no arbitrary rule excluding any nurse from joining the Pension Fund which Mr. Burdett has organised, nor are any "devoted women" "placed outside" its benefits, as our correspondent erroneously imagines. Hospital and private nurses will be able to join under favourable conditions, providing they are "noble and devoted women," as most of them no doubt are. We can assure our correspondent, and all whom it may concern, that "no class will be excluded under Mr. Burdett's scheme," which is organised on "a wide and far-reaching basis, worthy of the catholic appellation" of the National Pension Fund. If there are any other points which any reader is not clear about, we hope they will follow our correspondent's example, and send them to us without delay in writing.

